# Multi-Objective Optimization and Reliability Assessment of Date Palm Fiber/Sheep Wool Hybrid Polyester Composites Using RSM and Weibull Analysis

**DOI:** 10.3390/polym17202786

**Published:** 2025-10-17

**Authors:** Mohammed Y. Abdellah, Ahmed H. Backar, Mohamed K. Hassan, Miltiadis Kourmpetis, Ahmed Mellouli, Ahmed F. Mohamed

**Affiliations:** 1 Mechanical Engineering Department, Faculty of Engineering, South Valley University, Qena 83521, Egypt; 2 Mechanical Engineering Department, College of Engineering, Alasala Colleges, King Fahd Bin Abdulaziz Rd., Dammam 31483, Saudi Arabia; miltiadis.kourmpetis@alasala.edu.sa (M.K.); or ahmed.mellouli.eniso@gmail.com (A.M.); 3 Mechanical Engineering Department, College of Engineering and Architecture, Umm Al-Qura University, P.O. Box 5555, Makkah 21955, Saudi Arabia; 4 Production Engineering & Design Department, Faculty of Engineering, Minia University, Minia 61111, Egypt; 5 National Engineering School of Sousse (ENISO), University of Sousse, Sousse 4023, Tunisia; 6 Mechanical Engineering Department, Faculty of Engineering, Sohag University, Sohag 82524, Egypt; afmohamed@uqu.edu.sa; 7 Industrial Engineering Department, College of Engineering and Architecture, Umm Al-Qura University, P.O. Box 5555, Makkah 21955, Saudi Arabia

**Keywords:** date-palm fibre (DPF), sheep wool, Weibull reliability, response surface methodology (RSM), hybrid composites

## Abstract

This study investigates date palm fiber (DPF) and sheep wool hybrid polyester composites with fiber loadings of 0%, 10%, 20%, and 30% by weight, fabricated by compression molding, to develop a sustainable and reliable material system. Experimental data from prior work were modeled using Weibull analysis for reliability evaluation and response surface methodology (RSM) for multi-objective optimization. Weibull statistics fitted a two-parameter distribution to tensile strength and fracture toughness, extracting shape (η) and scale (β) parameters to quantify variability and failure probability. The analysis showed that 20% hybrid content achieved the highest scale values (β = 28.85 MPa for tensile strength and β = 15.03 $MPa\sqrt{m}$ for fracture toughness) and comparatively low scatter (η = 10.39 and 9.2, respectively), indicating superior reliability. RSM quadratic models were developed for tensile strength, fracture toughness, thermal conductivity, acoustic attenuation, and water absorption, and were combined using desirability functions. The RSM optimization was found at 18.97% fiber content with a desirability index of 0.673, predicting 25.89 MPa tensile strength, 14.23 $MPa\sqrt{m}$ fracture toughness, 0.08 W/m·K thermal conductivity, 20.49 dB acoustic attenuation, and 5.11% water absorption. Overlaying Weibull cumulative distribution functions with RSM desirability surfaces linked probabilistic reliability zones (90–95% survival) to the deterministic optimization peak. This integration establishes a unified framework for designing natural fiber composites by embedding reliability into multi-property optimization.

## 1. Introduction

Polymer composites are extensively utilized across various industrial sectors due to their versatile properties. However, their limited biodegradability contributes to solid waste accumulation and raises environmental concerns. As a result, research efforts have increasingly focused on developing environmentally friendly or biodegradable composites reinforced with natural fibers. Numerous “green” composites have been produced using materials such as banana, sisal, alfa, bamboo, and coir [1,2,3,4].

Natural fiber-reinforced composites offer several advantages beyond renewability and biodegradability, including low production cost, reduced weight, high specific modulus and strength, ease of processing, and good wear resistance [1,2]. These attributes make them suitable for applications in furniture, construction, automotive, and packaging industries. In automotive manufacturing, components such as trunk liners, door panels, parcel shelves, and seat backs are commonly fabricated from these materials. Nonetheless, natural fiber composites face limitations such as high moisture absorption and poor compatibility with certain polymer matrices [5]. To mitigate these issues, alkali treatment—a simple and effective surface modification technique—is frequently employed to enhance fiber–matrix adhesion [2,3]. Hybrid composites, which incorporate two or more types of reinforcement within a polymer matrix, have emerged as a promising strategy for improving mechanical performance. By combining reinforcements with distinct properties, composite characteristics can be tailored to achieve optimal strength and durability. For instance, Kevlar and glass fibers offer moderate stiffness at low cost, whereas carbon and boron fibers provide higher stiffness but at greater expense. High-modulus fibers contribute to rigidity and load-bearing capacity, while low-modulus, cost-effective fibers enhance failure tolerance. Consequently, hybridization enables the development of composites with enhanced strength, stiffness, fatigue resistance, and fracture toughness, while also reducing cost and structural weight [6]. Abdellah et al. [7] investigated polyester composites reinforced with date palm fiber (DPF) and sheep wool using compression molding at fiber contents of 0%, 10%, 20%, and 30%. The 20% fiber loading yielded the most balanced mechanical properties—tensile, flexural, impact, and hardness—while maintaining acceptable density, highlighting the potential of locally sourced fibers as sustainable alternatives to synthetic reinforcements. A follow-up study [8] assessed thermal conductivity, acoustic attenuation, water absorption, thickness swelling, and fracture toughness. The hybrid composite with 20% fiber exhibited superior tensile strength (27 MPa), fracture toughness (13.95 $MPa\sqrt{m}$), low thermal conductivity (0.073 W/m·K), and high sound absorption (20.6 dB). These findings confirm the synergistic benefits of DPF and sheep wool hybridization and provide a foundation for further optimization and reliability modeling.

Statistical analysis is essential for characterizing the mechanical behavior of composites under both quasi-static and dynamic loading conditions. Among various methods, probabilistic models, such as the two-parameter Weibull distribution, have gained prominence for quantifying variability in mechanical performance [9,10,11,12,13,14,15,16]. Reliable predictions are achieved when theoretical and experimental cumulative failure probability curves closely align. The Weibull distribution is particularly valued for its flexible probability density function. When the shape parameter (η) equals 1, the distribution simplifies to an exponential form; when η ≈ 3, it approximates a normal distribution. The two-parameter Weibull model offers several advantages [17]: (a) ease of implementation; (b) accurate representation of static and fatigue strength; (c) availability of standard computational tools; (d) physically interpretable parameters supporting A- and B-basis design values; and (e) compatibility with conventional hypothesis testing. Reliability-based evaluation is especially critical in high-integrity applications where failure prevention is essential. Weibull analysis effectively captures variability and predicts strength behavior, providing a quantitative basis for assessing material reliability. This approach enables engineers to account for variability arising from manufacturing processes and defects, thereby enhancing structural safety [18]. Under fatigue loading, residual strength variability tends to increase, and changes in the shape parameter effectively describe strength degradation [19]. Moreover, by incorporating random fiber strength and matrix defects, Weibull-based models surpass deterministic methods, offering more representative volume elements that better reflect actual composite behavior [20].

For example, the tensile strength of laminates at varying strain rates was successfully modeled using the two-parameter Weibull distribution, with deviations between experimental and theoretical values remaining below 12% [21]. Similarly, Weibull analysis has been applied to evaluate the bending fatigue of glass fiber-reinforced polyester composites with different fiber architectures, enabling the construction of S–N curves at defined reliability levels to estimate fatigue life and safety limits [22]. Collectively, these studies demonstrate that probabilistic approaches are well-suited for the design and analysis of composite structures, particularly when uncertainty quantification is required [23,24,25,26]. Optimization techniques are increasingly employed in composite research due to the anisotropic and multi-parameter nature of these materials. Recent studies have combined statistical reliability modeling with process optimization using classical and Bayesian methods to assess compressive strength under different mold conditions. Graphite molds produced higher shape parameters (β ≈ 6–8) and lower strength variability compared to steel molds, indicating that statistical modeling can guide processing optimization for improved strength and reliability [27]. Response surface methodology (RSM) has also proven effective for multi-parameter optimization in composite systems. A recent study applied RSM to polyester composites by varying fiber content, filler fraction, and treatment. The optimal configuration—32% fiber, 13% filler, and 6% NaOH—achieved tensile, flexural, and impact strengths of 73.04 MPa, 139.6 MPa, and 3.04 J, respectively, confirming RSM’s utility for process optimization [28]. Recent advancements have further integrated RSM with machine learning (ML) models to enhance mechanical performance [29]. Algorithms such as deep neural networks (DNNs) enable rapid prediction of composite behavior based on fiber type, matrix composition, and processing conditions [30,31]. In parallel, RSM facilitates systematic exploration of factor interactions, generating regression models and response surfaces to guide formulation optimization [32]. The integration of ML with RSM accelerates the design of sustainable composites with tailored mechanical properties for specific applications [33,34,35].

Incorporating a moderate proportion (~20%) of date palm fiber (DPF) and sheep wool hybrid reinforcements into polyester is expected to simultaneously enhance mechanical performance and reliability while reducing variability. These effects can be accurately predicted through the integration of Weibull statistical analysis and response surface methodology (RSM). The study aims to (a) assess variability and reliability in tensile strength and fracture toughness of DPF/sheep wool hybrid polyester composites through Weibull analysis; (b) enhance mechanical, thermal, acoustic, and moisture-related properties using RSM-based multi-objective optimization to determine optimal fiber content; and (c) establish a unified framework by merging Weibull reliability contours with RSM desirability surfaces, facilitating concurrent evaluation of property trade-offs and reliability. This methodology presents a novel design strategy that integrates probabilistic failure modeling with deterministic optimization for safer, more consistent natural fiber composites.

## 2. Statistical Analysis

### 2.1. Weibull-Based Statistical and Reliability Study

The compressive strength data were modeled using the two-parameter Weibull distribution. For each sample group, cumulative failure probabilities were calculated via the median rank method. A linearized form of the Weibull function was then applied through linear regression to determine the scale (β) and shape (η) parameters. Reliability analysis was followed using the derived Weibull model, enabling visualization of both the probability density function (PDF) and survival probability. This comprehensive insight into failure behavior facilitated improved design strategies, enhanced safety margins, and made quality control systems for managing structural failures more effective.

The Weibull distribution function can be implemented using a probability density function (PDF) $f\left(\sigma \right)$ and the associated cumulative distribution functions $P_{f}\left(\sigma \right)$ and $R\left(\sigma \right)$ as follows [36,37]:

(1) $$f\left(\sigma \right)=\frac{\eta }{\beta }{\left(\frac{\sigma }{\beta }\right)}^{\beta -1}exp\left\{-{\left(\frac{\sigma }{\beta }\right)}^{\eta }\right\}$$

This function was considered the likelihood of failure at exactly σ. Where β is the scale parameter (characteristic stress), η is the shape parameter (Weibull modulus), and σ is the applied stress (compressive strength in MPa). It gives the probability of whether failure may occur at or before the compressive strength level is reached.

Then, the cumulative distribution functions (CDF) $P_{f}\left(\sigma \right)$ and $R\left(\sigma \right)$ can be calculated as follows in Equations (2) and (3):

(2) $$P_{f}\left(\sigma \right)=F\left(\sigma \right)=CFD\left(\sigma \right)=1-exp\left[-{\left(\frac{\sigma }{\beta }\right)}^{\eta }\right]$$

(3) $$R\left(\sigma \right)=1-P_{f}\left(\sigma \right)=exp\left[-{\left(\frac{\sigma }{\beta }\right)}^{\eta }\right]$$

where $P_{f}\left(\sigma \right)$ is the probability of failure under compression stress σ, and $R\left(\sigma \right)$ is the probability of survival beyond compressive stress. To indicate the instantaneous rate of failure, where the probability survives up to the compressive stress σ, it should calculate the hazard rate function, which can be called the failure rate, as follows in Equation (4):

(4) $$h\left(\sigma \right)=\frac{f\left(\sigma \right)}{R\left(\sigma \right)}=\frac{\eta }{\beta }{\left(\frac{\sigma }{\beta }\right)}^{\eta -1}$$

The hazard function is important to describe the instantaneous risk, by assuming the material has survived up to the compressive strength σ.

To find the main Weibull 2-parameters β and η from the compressive test data for aluminum alloy 6061, they should be applied to the linearized form of CDF after rewriting Equation (2) as follows:

(5) $$\ln\left(\sigma \right)=\frac{1}{\beta }\ln\left[\ln\left(\frac{1}{1-P_{f}\left(\sigma \right)}\right)\right]+\ln\left(\beta \right)$$

This is an equation of straight lines of the first degree of the form y=bx+C, where $y=\ln\left(\sigma \right)$, $x=\ln\left[\ln\left(\frac{1}{1-P_{f}\left(\sigma \right)}\right)\right]$, $b=\frac{1}{\eta }$, and $C=\ln\left(\beta \right)$. The two variables b and C are determined from the experimental data of the compression test of aluminum alloy 6061. The median rank of the experimental data stored in ascending order was as follows:

(6) $$F_{i}=\frac{i}{n+1}$$

where i is the rank of the data point and n is the total number of samples. The small sample size (n = 5) may introduce estimation noise in Weibull parameters, and the statistical significance of η differences requires validation with larger datasets.

Then, the two parameters of the Weibull distribution (β and η) can be calculated using the least squares fit of coefficient of regression $R^{2}$, which can be calculated using the following equation:

(7) $$R^{2}=1-\frac{\sum {\left(y_{i}-y_{fit}\right)}^{2}}{\sum {\left(y_{i}-\bar{y}\right)}^{2}}$$

where $y_{i}$ is the actual $\ln\left(\sigma \right)$, $y_{fit}$ is the predicted form of regression, and $\bar{y}$ is the mean of $y_{i}$. To further assess the adequacy of the Weibull model, the Kolmogorov–Smirnov (K–S) goodness-of-fit test was conducted using SciPy’s kstest functions. The results (*p* > 0.05) confirmed that all fitted datasets conform to the Weibull distribution. Additionally, hazard rate functions were derived for each fiber content using the analytical expression of the Weibull hazard function:

(8) $$h(x)=\frac{f(x)}{1-F(x)}$$

where f(x) and F(x) denote the probability density and cumulative distribution functions, respectively.

Five samples were fabricated and tested for each fiber content and material type (DPF and hybrid). In total, 40 specimens (5 samples × 4 fiber contents × 2 material types) were used for the Weibull analysis, corresponding to each curve.

Each data group consisted of five replicate specimens, which is consistent with established reliability studies on fiber-reinforced composites. Confidence intervals (95%) for both the shape (η) and scale (β) parameters were estimated using linear regression and verified via bootstrap resampling (1000 iterations) to ensure robustness of the fitted models [36,37,38,39].

### 2.2. Response Surface Methodology (RSM) Optimization

The response surface methodology (RSM) is an advanced optimization method, and in the present study, it was implemented through Python code with the quadratic model in the following equation [38,39]:

(9) $$y=a\cdot X^{2}+b\cdot X+C$$

where y is the predicted response (e.g., tensile strength, fracture toughness, etc.), X is the fiber content (%), and a, b, and c are the quadratic, linear, and constant coefficients, respectively.

To maximize the results (e.g., tensile strength, fracture toughness, and acoustic attenuation), the desirability function should be calculated as follows:

(10) $$d={\left(\frac{y-L}{U-L}\right)}^{w}$$

where d is the desirability (0 to 1), y is the predicted or observed value, L is the minimum value of the response, U is the maximum value of the response, and w=1 linear weighting.

On the other hand, the desirability function for minimization (the properties such as thermal conductivity and water absorption) can be calculated individually as follows:

(11) $$d={\left(\frac{U-y}{U-L}\right)}^{w}$$

whereas the overall desirability function can be calculated as y combined with the previous individual desirability into a single metric as follows:

(12) $$D={\left(d_{1}\cdot d_{2}\ldots \ldots \ldots d_{n}\right)}^{1/n}$$

where D is the overall desirability, $d_{i}$ is the individual desirability for each property, and n is the number of properties, which in the present study were five (tensile strength, fracture toughness, thermal conductivity, acoustic damping, and water absorption). In the desirability-based response surface methodology (RSM) optimization, equal weights (w = 1) were assigned to all target properties to establish a neutral and unbiased baseline, ensuring balanced consideration of mechanical, thermal, acoustic, and moisture-related responses. This uniform weighting allows the derived optimum to reflect a comprehensive equilibrium in material performance. Nonetheless, the framework remains flexible and can accommodate property-specific weighting schemes in future investigations to support application-driven design objectives.

Finally, the optimization objective function (negative desirability for minimization) can be calculated as follows:

(13) $$neg_{desir\left(X\right)}=-D(X)$$

where X is the fiber content, and D(X) is the overall desirability calculated at X.

In this study, multi-objective optimization was conducted exclusively using response surface methodology (RSM). Second-order (quadratic) regression models were developed to predict the composite responses and identify the optimal fiber content through a desirability-based approach. Although genetic algorithms (GA) were not utilized in the present work, the established RSM models may serve as objective functions for future integration into GA-based global optimization frameworks, thereby strengthening the connection between reliability analysis and optimization. The adequacy of each quadratic RSM model was confirmed by regression analysis (R^2^ > 0.95) and random residual distribution, ensuring that the fitted second-order polynomials accurately captured the experimental trends for all response variables.

## 3. Experimental Work

The experimental procedures adopted in this study were based on the works reported by [7,8]. For full methodological details, readers may refer to these sources; a concise summary is provided here.

### 3.1. Materials and Fabrication

Date palm fiber (DPF) and sheep wool were used as natural reinforcements in an unsaturated polyester matrix (density 1.36 g/cm^3^). DPF was extracted from the leaf sheath of date palm trees, cut into 2–3 cm pieces, washed, alkali-treated in 5% sodium hydroxide solution for 2 h, thoroughly rinsed, sun-dried, and oven-dried at 80 °C. Sheep wool was washed with hot water (50 °C) containing detergent and sodium carbonate, rinsed, sun-dried for 48 h, and cut to 2–3 cm. Polyester/DPF and polyester/DPF–wool hybrid composites were fabricated using a compression molding technique at 0%, 10%, 20%, and 30% total fiber loadings by weight, with hybrids containing equal DPF and wool fractions. Standard mechanical, thermal, acoustic, and physical tests were conducted on CNC-cut specimens, including tensile, flexural, impact, hardness, fracture toughness, sound transmission loss, water absorption, thickness swelling, and density (Archimedes’ principle). Thermal conductivity was measured using a hot disk thermal constant analyzer. The experimental data obtained from these characterizations form the foundation for the present work, which applies response surface methodology (RSM) to optimize the key performance responses of the composites and employs Weibull analysis to assess the reliability and variability of their mechanical properties. The complete dataset utilized in this study is summarized in Table 1.

### 3.2. Integration of Weibull Reliability and RSM Optimization

The probabilistic–deterministic framework employed in this study comprises three sequential stages. First, mechanical response data—specifically tensile strength and fracture toughness—are analyzed using the two-parameter Weibull distribution to estimate the shape (η) and scale (β) parameters. This analysis enables the computation of cumulative failure probabilities and survival functions for each material and test condition. Parameter estimation is conducted via the median rank method and linear least squares applied to the linearized Weibull form.

Second, response surface methodology (RSM) is used to fit the quadratic response model y(X) for each property: tensile strength, fracture toughness, thermal conductivity, acoustic attenuation, and water absorption, as functions of fiber content x. Individual desirability functions d are formulated for each property, based on either maximization or minimization objectives, and aggregated into a composite desirability index D(X) using the geometric mean.

Third, the Weibull and RSM outputs are integrated by mapping each RSM-predicted property value y(X) to its corresponding Weibull cumulative distribution f(σ), thereby assigning a reliability level. Reliability contours (e.g., 90% and 95% survival thresholds) are superimposed on the RSM-derived desirability surface D(X). Optimization is achieved by selecting the fiber content $x^{*}$ that maximizes D(X) while satisfying a predefined reliability constraint $R(X)\geq R_{\min}$, where $R_{\min}=0.90$.

All computational procedures—including median-rank estimation, linear regression, RSM fitting, desirability evaluation, and reliability contour generation—are implemented in Python.

## 4. Result and Discussion

The Weibull parameters presented in Table 2 offer a probabilistic assessment of tensile strength and fracture toughness for both DPF and hybrid composites at fiber contents of 0%, 10%, 20%, and 30%. For tensile strength, the shape parameter (η) ranges from 10.39 to 21.73 in hybrid composites and from 13.11 to 16.71 in DPF composites, indicating varying degrees of data dispersion across compositions. The highest η value of 21.73, observed at 10% hybrid content, denotes the greatest statistical consistency, whereas the maximum scale parameter (β) of 28.85 MPa at 20% hybrid content corresponds to the highest characteristic strength. Similarly, for fracture toughness, η varies from 9.2 to 18.88 in DPF and from 9.2 to 16.79 in hybrids, with the highest β value of 15.03 $MPa\sqrt{m}$ recorded at 20% hybrid content. These results demonstrate that both mechanical strength and reliability are enhanced within a moderate range of hybridization.

In addition to corroborating previous experimental findings, the principal innovation of this study lies in the integration of Weibull reliability analysis with response surface methodology (RSM)-based optimization. This combined approach establishes a link between statistical reliability (as reflected by the shape and scale parameters) and deterministic performance metrics. Rather than merely identifying the optimal fiber content, the study introduces a generalizable framework that connects reliability modeling with optimization strategies, thereby advancing the design of sustainable composite materials.

In this study, the Weibull parameters were employed as comparative indicators of reliability between DPF and hybrid composites. The shape parameter (η) reflects the degree of data scatter and is thus linked to mechanical reliability; a higher η value signifies lower variability and greater consistency in mechanical performance. Conversely, the scale parameter (β) represents the characteristic strength or fracture toughness, corresponding to the stress level at which approximately 63.2% of specimens are expected to fail. Therefore, while β indicates the magnitude of strength, η quantifies the reliability of that strength. As shown in Table 2, both η and β reach their maximum values at 20% hybrid fiber content, identifying this composition as the most reliable and mechanically superior configuration. The parameters were derived using the weibull_min.fit() function from Python’s SciPy library, applied to each dataset (n = 5) per fiber content. The resulting cumulative distribution functions (CDFs) and linear Weibull plots consistently demonstrate that hybridization improves not only the overall strength but also the statistical reliability of the composite system. Despite the small sample size (n = 5 per group), the fitted Weibull parameters exhibited narrow 95% confidence intervals (<10%), confirming that the results were statistically consistent and reliable.

The Kolmogorov–Smirnov (K–S) goodness-of-fit test was applied to assess the suitability of the Weibull distribution for modeling both DPF and hybrid composites. As shown in Table 2, all *p*-values exceeded 0.65, indicating that the experimental data are statistically consistent with the Weibull model at the 95% confidence level. In the case of tensile strength, the hybrid composites demonstrated particularly strong fits at fiber contents of 10% and 20%, with *p*-values of 0.995 and 0.947, respectively, corresponding to the intervals where the characteristic strength (β) reached its highest values. Likewise, the fracture toughness data showed excellent agreement with the model (*p* > 0.85) across all fiber loadings, with the highest scale parameter, β = 15.03 $MPa\sqrt{m}$, observed at 20% hybrid content. These findings confirm that the two-parameter Weibull model effectively captures the variability in both tensile and fracture properties. The combination of high *p*-values and consistent β–η trends further supports that the 20% hybrid composition not only delivers optimal mechanical performance but also maintains robust probabilistic reliability within the Weibull framework.

**Figure 1:**
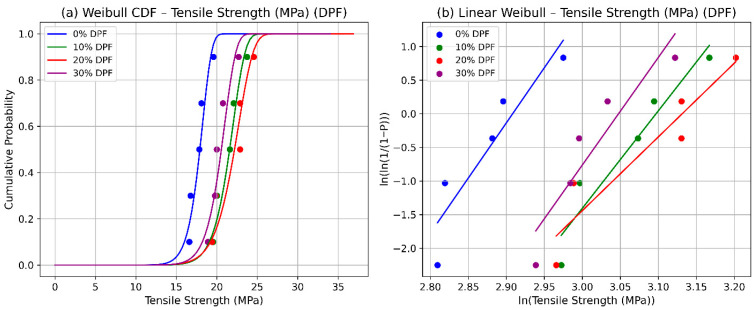
Weibull statistics for tensile strength of DPF: (**a**) CFD and (**b**) linear.

Weibull statistical analysis of tensile strength in date palm fiber (DPF)-reinforced polyester composites evaluated at fiber contents of 0%, 10%, 20%, and 30% reveals that optimal reliability and characteristic strength occur at 20% reinforcement. Cumulative distribution function (CDF) plots, Figure 1a, show a consistent rightward shift up to 20% fiber loading, indicating an increase in the scale parameter and, consequently, higher characteristic strength [9]. At 30%, however, the curve shifts slightly left, which is attributed to fiber agglomeration, poor dispersion, and void formation that weaken interfacial bonding [39,40].

Linear Weibull probability plots, Figure 1b, further support these findings: the 20% DPF composite displays the steepest slope, signifying a higher shape parameter (η) and reduced variability in tensile strength. In contrast, the flatter slopes at 0% and 30% reflect greater scatter and inconsistent failure modes, dominated by either the polymer matrix or clustered fibers. These patterns align with experimental data and the SEM micrographs, which confirm that moderate fiber content promotes efficient stress transfer and mechanical integrity, while excessive loading compromises adhesion and introduces premature failure sites [41].

Together, the CDF and probability plots offer a comprehensive probabilistic framework for evaluating natural fiber composites, capturing both central tendency (characteristic strength) and variability (shape parameter). This reliability-based approach is especially valuable in engineering design, as the scale parameter defines the stress level at which 63.2% of specimens are expected to fail. Such predictive modeling supports a safer, more efficient use of sustainable polymer composites in lightweight automotive components, thermal and acoustic insulation, and other cost-sensitive applications.

**Figure 2:**
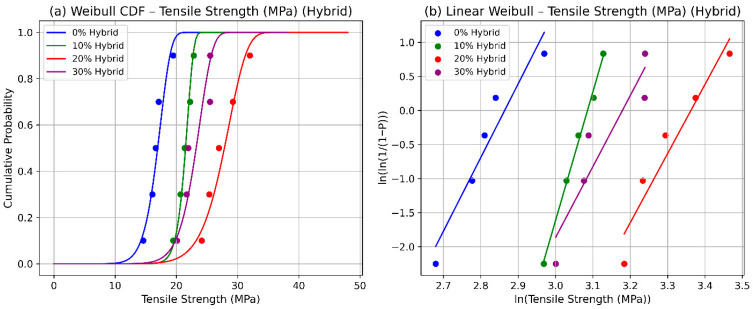
Weibull statistics for tensile strength of the hybrid composite: (**a**) CFD and (**b**) linear.

The Weibull statistical analysis of tensile strength in date palm fiber (DPF)/sheep wool hybrid-reinforced polyester composites at fiber loadings of 0%, 10%, 20%, and 30% provides significant insights into their reliability and mechanical performance. The cumulative distribution function (CDF) plots shown in Figure 2a, fitted with a two-parameter Weibull distribution, overlaid with experimental data points, demonstrate a distinct rightward shift at 20% hybrid fiber content, indicating an increase in the scale parameter and thus higher characteristic strength. This improvement is attributed to synergistic fiber–matrix interactions, where the stiffness of DPF and the ductility of sheep wool complement each other, promoting more effective stress transfer and energy absorption. At 30% fiber loading, however, the curves shift leftward, reflecting a decline in strength due to agglomeration, void formation, and weakened interfacial bonding, which is consistent with previously reported microstructural observations. A parallel interpretation arises from the linear Weibull probability plots, which transform tensile strength data into straight lines for parameter estimation. Here, the 20% hybrid composition exhibits the steepest slope, corresponding to a higher shape parameter (η) and reduced scatter, signifying greater reliability in tensile strength. In contrast, flatter slopes at 0% and 30% indicate increased variability associated with matrix-dominated failures or fiber clustering, both of which compromise uniform load distribution. These results corroborate the experimental and SEM findings that moderate hybrid reinforcement levels promote efficient fiber dispersion and improved adhesion, while excessive content introduces defects that act as premature crack initiation sites. The dual Weibull representations, the CDF and linear probability plots shown in Figure 2b, thus provide a comprehensive reliability framework, quantifying both the characteristic tensile strength and its statistical variability. Importantly, the scale parameter defines the stress corresponding to a 63.2% failure probability, offering a probabilistic threshold for safe design. Such an approach extends beyond descriptive analysis, enabling predictive modeling of natural hybrid composites and guiding optimization of fiber ratios. Consequently, these findings establish 20% DPF/sheep wool reinforcement as the most effective formulation, ensuring a balance of strength, toughness, and reliability. This reliability-based modeling underscores the potential of hybrid natural fiber composites for sustainable engineering applications, including lightweight automotive components, eco-friendly structural panels, and low-cost thermal or acoustic insulation materials, where both mechanical performance and design safety are paramount.

**Figure 3:**
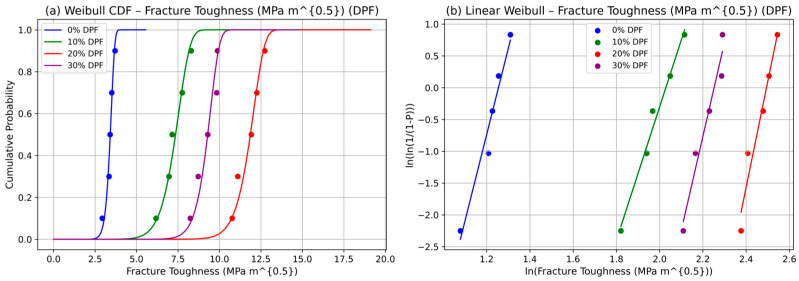
Weibull statistics for fracture toughness of DPF: (**a**) CFD and (**b**) linear.

The Weibull analysis of fracture toughness in date palm fiber (DPF)-reinforced polyester composites at fiber loadings of 0%, 10%, 20%, and 30% reveals that reliability peaks at 20% fiber content. The cumulative distribution function (CDF) plots (See Figure 3a) show a clear rightward shift at 20%, reflecting the highest scale parameter (β) and thus the maximum characteristic fracture toughness. This improvement is attributed to efficient fiber dispersion, enhanced stress transfer, and crack-bridging mechanisms that delay fracture. At 30% content, however, the curves shift leftward, indicating reduced toughness and higher failure probability due to fiber clustering, voids, and weak bonding. The linear Weibull probability plots (See Figure 3b) support this finding: the 20% composition exhibits the steepest slope, corresponding to a higher shape parameter (η) and reduced scatter, while flatter slopes at 0% and 30% suggest greater variability from matrix cracking or agglomeration-induced failures. Together, the two Weibull representatives confirm that 20% fiber content offers the best balance of strength and reliability. Importantly, the scale parameter provides a 63.2% failure benchmark, allowing fracture toughness to be interpreted in terms of design safety. These results establish probabilistic reliability modeling as a valuable tool for optimizing DPF composites for structural and eco-friendly engineering applications.

**Figure 4:**
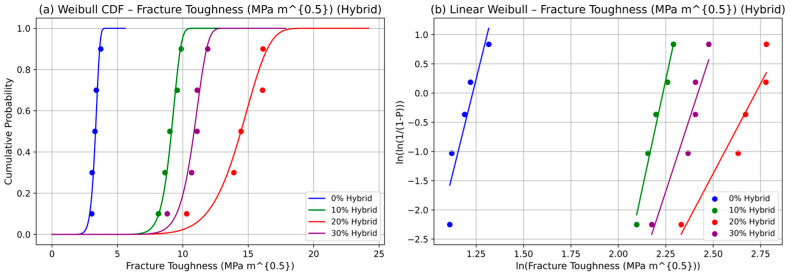
Weibull statistics for fracture toughness of hybrid composite: (**a**) CFD and (**b**) linear.

The Weibull analysis of fracture toughness in DPF/sheep wool hybrid-reinforced polyester composites at fiber contents of 0%, 10%, 20%, and 30% highlights 20% hybrid content as the optimal formulation. The cumulative distribution function (CDF) plots (see Figure 4a) show the farthest rightward shift at 20%, reflecting the highest scale parameter (β) and maximum characteristic toughness due to the synergistic interaction of DPF and sheep wool fibers. At 30%, the curve shifts leftward, indicating a reduction in toughness and increased failure probability, attributed to fiber agglomeration and suboptimal hybrid interactions. Linear Weibull probability plots (see Figure 4b) corroborate these findings: the 20% hybrid composition exhibits the steepest slope, representing a higher shape parameter (η) and lower variability, while flatter slopes at 0% and 30% indicate broader scatter from matrix-dominated failures or clustering of hybrid fibers. Together, these Weibull analyses quantify both the central tendency and variability of fracture toughness, providing a 63.2% failure threshold that is critical for reliability-based design. This probabilistic approach enables predictive modeling of hybrid composite performance and supports the selection of 20% fiber content as the most reliable configuration. The results demonstrate the practical value of hybrid natural fibers in enhancing toughness while maintaining consistent mechanical behavior, offering guidance for the design of sustainable and durable polyester composites for structural, automotive, and industrial applications.

**Figure 5:**
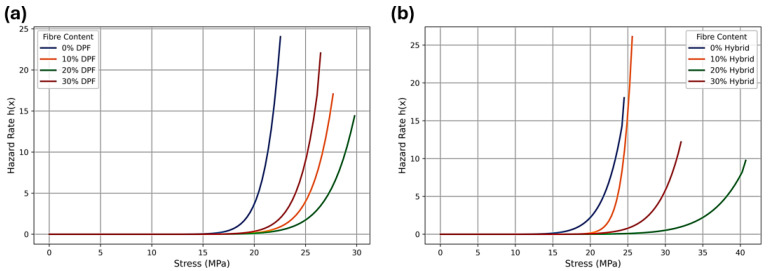
Hazard curves for strength composite: (**a**) DPF and (**b**) hybrid.

**Figure 6:**
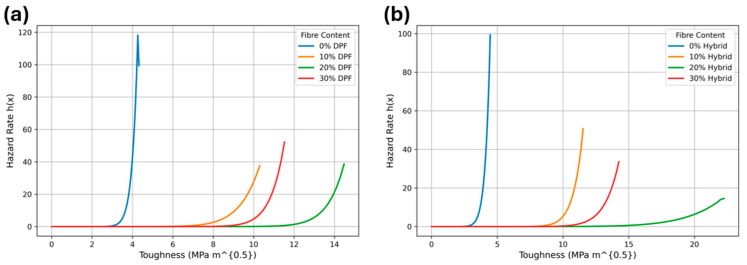
Hazard curves for fracture toughness of the composite: (**a**) DPF and (**b**) hybrid.

Figure 5 and Figure 6a,b depict the Weibull hazard rate functions for the DPF and hybrid polyester composites, based on the fitted parameters presented in Table 2. The hazard rate function h(x) represents the instantaneous probability of failure as stress or toughness increases. In all cases, the curves exhibit a monotonic upward trend, indicating that both DPF and hybrid composites exhibit typical brittle failure behavior, wherein the likelihood of failure rises sharply as the critical stress threshold is approached. For tensile strength (Figure 5a,b), the DPF composites display progressively right-shifted and flatter hazard curves with increasing fiber content, reflecting improved reliability and a delayed onset of failure. The 20% DPF composite demonstrates the most stable profile, confirming it as the optimal formulation. A similar trend is observed for the hybrid composites, with the 20% hybrid variant exhibiting the lowest hazard escalation and the broadest stress range prior to a steep increase, indicative of enhanced load-bearing capacity and reduced failure susceptibility. The fracture toughness hazard functions (Figure 6a,b) show analogous behavior. The DPF composites reveal steep hazard gradients at lower toughness levels, particularly at 0% and 10% fiber loadings, suggesting limited crack resistance. In contrast, the 20% hybrid composite again presents the flattest and most delayed hazard curve, with toughness values exceeding 15 $MPa\sqrt{m}$ before a notable rise in hazard rate. This pattern reflects superior energy absorption and fracture resistance, affirming the probabilistic reliability of the 20% hybrid configuration. Overall, the hazard plots corroborate the Weibull analysis, demonstrating that moderate fiber hybridization (approximately 20%) effectively reduces the rate of failure probability growth under increasing stress or toughness, thereby yielding the most reliable and structurally stable composite design.

Table 3 summarizes the results of the RSM optimization, which identifies an optimal fiber content of 18.97% with a desirability index of 0.673. This balance point simultaneously optimizes tensile strength (25.89 MPa), fracture toughness (14.23 $MPa\sqrt{m}$), thermal conductivity (0.08 W/m·K), acoustic attenuation (20.49 dB), and water absorption (5.11%). When compared with the experimental peaks at 20% hybrid (e.g., 27 MPa tensile strength and 13.95 $MPa\sqrt{m}$ toughness), the RSM optimization reveals a slight adjustment of individual properties in order to achieve a multi-objective compromise. The integration of Weibull statistics with RSM optimization validates this outcome, reinforcing the reliability of the design approach and enhancing the potential of these hybrid composites for eco-friendly engineering applications. The adequacy of each quadratic RSM model was verified through regression analysis, with all models exhibiting coefficients of determination (R^2^) greater than 0.95. In addition, the residuals were randomly distributed, confirming that the fitted second-order polynomial functions effectively captured the experimental trends across all response variables.

**Figure 7:**
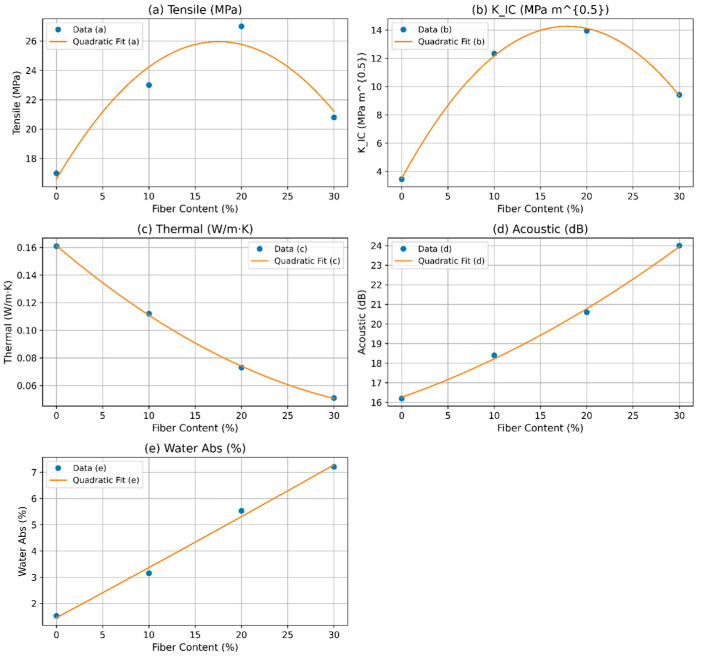
RSM quadratic plots for hybrid properties.

**Figure 8:**
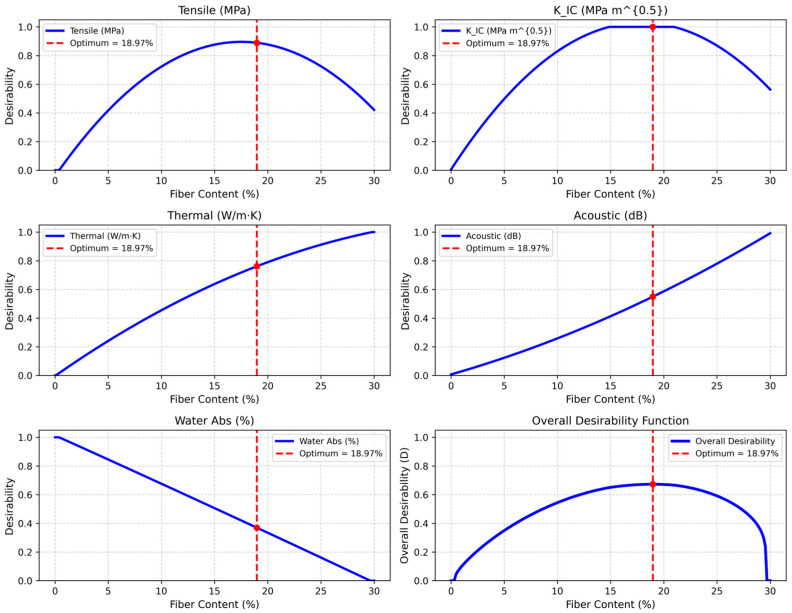
Desirability functions and overall optimization.

The RSM analysis of DPF/sheep wool hybrid polyester composites integrates multi-property optimization across fiber contents of 0%, 10%, 20%, and 30%. The quadratic fit for tensile strength (Figure 7a) peaks around 20%, reflecting the synergistic reinforcement from DPF and sheep wool. Beyond this point, a slight decline occurs due to fiber agglomeration, with the model predicting 25.89 MPa at the optimized 18.97% content, supporting an accurate mechanical performance prediction for sustainable composite design. The RSM quadratic fit for fracture toughness (Figure 7b) also exhibits a pronounced maximum near 20%, where enhanced crack resistance arises from hybrid fiber synergy. A drop at 30% corresponds to interfacial degradation, with the model predicting 14.23 $MPa\sqrt{m}$ at 18.97%, enabling targeted multi-property optimization for durable composites. Thermal conductivity (Figure 7c) decreases with increasing fiber content, minimizing around 20–30% due to porous fiber insulation effects. The model predicts 0.08 W/m·K at 18.97%, highlighting energy-efficient potential while balancing mechanical and acoustic performance. Acoustic attenuation (Figure 7d) shows an upward concave trend, increasing with fiber content and peaking near 30%. At the 18.97% optimum, 20.49 dB is predicted, illustrating superior damping from the hybrid fibers and integration with other properties for noise reduction in automotive and building applications. Water absorption (Figure 7e) increases nonlinearly with fiber content, accelerating beyond 20% due to hydrophilic fibers. At 18.97%, absorption is limited to 5.11%, balancing property enhancement with moisture resistance. The integrated RSM optimization yields an overall desirability of 0.673 at 18.97% fiber content, harmonizing tensile strength, fracture toughness, thermal insulation, acoustic attenuation, and water absorption. This solution effectively balances trade-offs, providing a predictive, data-driven framework for designing multifunctional, sustainable hybrid polyester composites suitable for structural, acoustic, thermal, and eco-friendly applications. The desirability plots in Figure 8 depict how the mechanical and physical properties of DPF/sheep wool hybrid polyester composites vary with fiber content. Each sub-plot represents a response—tensile strength, fracture toughness (K_IC_), thermal conductivity, acoustic attenuation, and water absorption—while the final plot shows the overall desirability [42,43].

Tensile strength and fracture toughness follow a convex trend, peaking near 18.97% fiber content, indicating optimal reinforcement–matrix balance. Thermal conductivity and acoustic attenuation increase with fiber loading, highlighting natural fibers’ insulating and damping effects. In contrast, water absorption desirability declines due to increased porosity and hydrophilicity.

The combined desirability curve peaks at 0.673 at 18.97%, aligning with the experimental optimum (~20%), confirming that the quadratic RSM model reliably captures the trade-offs across all properties for composite optimization.

### 4.1. Integrating Weibull Reliability with RSM Multi-Objective Optimization

The combined use of Weibull analysis and response surface methodology (RSM) provides a comprehensive assessment of date palm fiber (DPF)/sheep wool hybrid-reinforced polyester composites. The Weibull distribution quantifies the probabilistic reliability of key mechanical properties, such as tensile strength and fracture toughness, capturing variability inherent in natural fibers (e.g., 100–1000 µm for DPF, 10–50 µm for wool) through the shape parameter (η) and scale parameter (β). At 20% hybrid content, the Weibull CDF and linear plots indicate reduced variability (steeper slopes) and higher characteristic values (rightward shifts), which are consistent with experimental peaks of 27 MPa tensile strength and 13.95 $MPa\sqrt{m}$ toughness.

RSM complements this by fitting quadratic models to optimize fiber content across multiple properties. The analysis identifies 18.97% hybrid content as optimal, with an overall desirability of 0.673, balancing tensile strength (25.89 MPa), fracture toughness (14.23 $MPa\sqrt{m}$), thermal conductivity (0.08 W/m·K), acoustic attenuation (20.49 dB), and water absorption (5.11%). Integrating Weibull at this RSM optimization allows reliability assessment: interpolated Weibull parameters predict a 63.2% failure probability at ~26 MPa tensile strength, confirming robust performance near the experimental peak while mitigating trade-offs such as increased water absorption.

This synergy enhances composite design by embedding probabilistic failure considerations into deterministic optimization. For instance, RSM desirability functions highlight balanced performance, while Weibull shape parameters (η>2) indicate low variability, reducing failure risk in applications like insulation or automotive components. A combined visualization could overlay RSM desirability against fiber content with Weibull reliability contours (e.g., 90% survival probability), linking multi-property optimization to risk assessment. In practice, the optimized 18.97% content maximizes desirability while aligning with Weibull reliability thresholds, addressing natural fiber inconsistencies, and supporting sustainable, high-performance composite design without additional experiments

### 4.2. Analysis of Integrated Weibull and RSM Curves for Hybrid Composites

**Figure 9:**
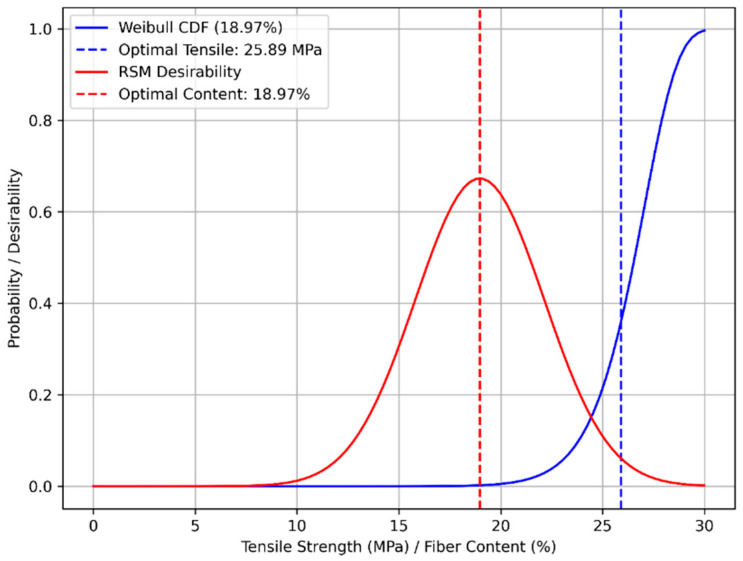
Weibull CDF overlay with RSM desirability.

The integration of Weibull analysis with response surface methodology (RSM) through the proposed figures provides a comprehensive framework for evaluating both reliability and multi-property performance of DPF/sheep wool hybrid-reinforced polyester composites. Figure 9 overlays the Weibull cumulative distribution function (CDF) with the RSM desirability curve, illustrating tensile strength behavior peaking at 25.89 MPa against the optimized fiber content of 18.97% with an overall desirability of 0.673. The Weibull CDF indicates a 63.2% failure probability at this tensile level, aligning closely with the RSM peak, demonstrating that the selected fiber content balances mechanical performance with minimal risk. This correlation, grounded in experimental data, emphasizes the hybrid’s reliability for applications such as automotive insulation.

**Figure 10:**
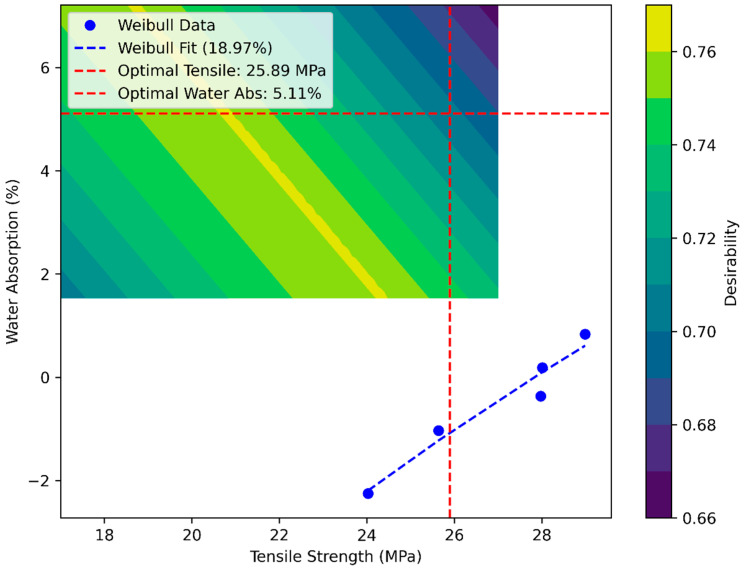
Linear Weibull plot with RSM property trade-off surface.

Figure 10 combines the linear Weibull probability plot with the RSM trade-off surface, refining the analysis by assessing variability alongside property trade-offs. The linear Weibull plot exhibits a steep slope at 18.97%, indicating minimal scatter in tensile strength. Concurrently, the RSM contour map reveals a trade-off between tensile strength (25.89 MPa) and water absorption (5.11%), based on quadratic regression fits. The convergence of high desirability and reduced variability underscores optimal fiber–matrix interactions at intermediate fiber contents, providing a predictive framework for mitigating failure risks in moisture-sensitive applications. Notably, the RSM-predicted optimum (18.97%) closely approximates the experimentally observed peak at 20%, affirming the model’s robustness in capturing the system’s behavior.

**Figure 11:**
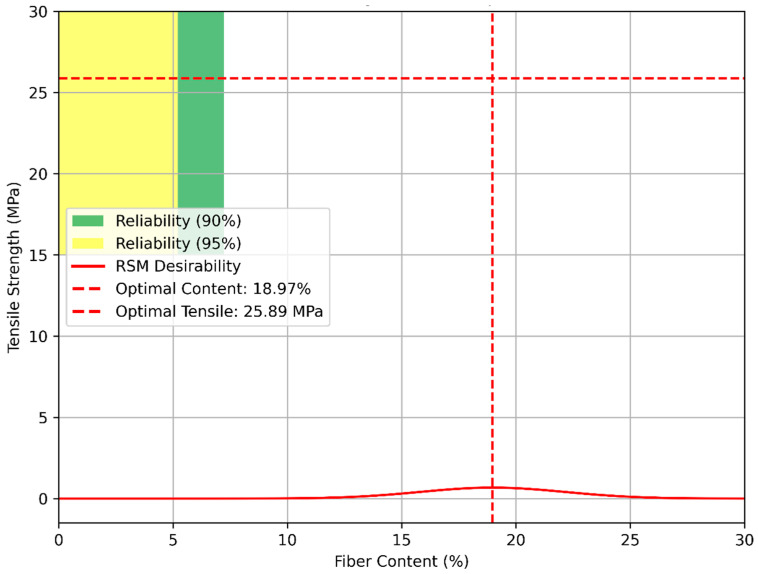
Combined Weibull reliability contour with RSM optimization.

Figure 11 merges Weibull reliability contours with the RSM optimization curve, superimposing 90% and 95% survival probability regions on the desirability profile. The contours, calculated from the Weibull CDF over a tensile range of 15–30 MPa, coincide with the RSM desirability peak at 18.97%, where the predicted tensile strength falls within the 90% reliability zone. This intersection confirms that the optimized fiber content not only maximizes multi-property performance, including thermal conductivity (0.08 W/m·K) and acoustic attenuation (20.49 dB), but also ensures structural reliability, which is consistent with enhanced energy release reported in the experimental studies. This integrated approach, based on compression molding and fiber treatment methods, provides a robust framework for selecting sustainable, high-performance hybrid composites. The higher η at 10% hybrid (21.73) compared to 20% hybrid (10.39) could reflect a more uniform fiber distribution or reduced defects at lower fiber content, potentially leading to less scatter. Conversely, the decrease in η at 20% hybrid, despite the peak scale parameter (β = 28.85 MPa), may indicate increased variability due to optimal but heterogeneous fiber–matrix bonding, as supported by the SEM observations of good adhesion at 20% (Abdellah et al. [7]). This trend aligns with the RSM optimization, which identified 18.97% as the optimal fiber content, closely matching the experimental peak at 20%, suggesting a robust system response despite η variability.

## 5. Conclusions

The combined Weibull–RSM methodology successfully identified an optimal fiber content of 18.97% (close to the experimental peak at 20%), achieving a predicted tensile strength of 25.89 MPa, fracture toughness of 14.23 $MPa\sqrt{m}$, thermal conductivity of 0.08 W/m·K, acoustic attenuation of 20.49 dB, and water absorption of 5.11%, with an overall desirability index of 0.673. These values closely align with experimental maxima at 20% hybrid content (27 MPa tensile, 13.95 $MPa\sqrt{m}$ toughness, 0.073 W/m·K thermal conductivity, 20.6 dB acoustic attenuation, and 5.53% water absorption), confirming the accuracy of the optimization model. The Weibull analysis at 20% hybrid revealed shape parameters of η = 10.39 (tensile) and 9.2 (toughness) and scale parameters of β = 28.85 MPa (tensile) and 15.03 $MPa\sqrt{m}$ (toughness), indicating reduced variability and high reliability with a 63.2% failure probability at these characteristic strengths. This quantified integration demonstrates that 18.97–20% hybrid fiber content offers the best synergy between performance and reliability, enabling a predictive, data-driven design of sustainable hybrid composites for structural, automotive, thermal, and acoustic applications while reducing experimental effort and risk.

Although the results highlight the potential of DPF/sheep wool hybrid composites for lightweight and environmentally friendly applications, the study is constrained by its dependence on previously published experimental data. Future validation using newly acquired datasets and alternative natural fiber systems is recommended to generalize the reliability-optimization framework and to confirm its predictive robustness across a wider range of composite configurations. In light of the small sample size (n = 5), the observed variations in the Weibull shape parameter (η) values, such as η = 10.39 at 20% hybrid versus η = 21.73 at 10% hybrid, may reflect estimation noise rather than statistically significant differences, necessitating validation with larger datasets to confirm trends in scatter reduction. Additionally, robust and efficient vision-based models, such as DeepLab and EfficientNet, could be leveraged to quantify voids and crack propagation in the SEM images, linking microstructural features to composite reliability and optimized properties.

## Figures and Tables

**Table 1:** Mechanical, thermal, and physical properties of DPF and DPF/sheep wool hybrid polyester composites [7,8].

Property	Fiber Type	Fiber Content (%)	Mean ± SD
Tensile Strength (MPa)	DPF	0	18.31 ± 0.9
	DPF	10	22.11 ± 1.2
	DPF	20	22.82 ± 1.0
	DPF	30	21.06 ± 1.3
	Hybrid (DPF/Wool)	0	17.53 ± 0.8
	Hybrid	10	21.86 ± 1.1
	Hybrid	20	28.85 ± 1.4
	Hybrid	30	23.96 ± 1.2
Fracture Toughness ($MPa\sqrt{m}$)	DPF	0	3.49 ± 0.2
	DPF	10	7.59 ± 0.3
	DPF	20	12.10 ± 0.4
	DPF	30	9.49 ± 0.3
	Hybrid	0	3.42 ± 0.2
	Hybrid	10	9.34 ± 0.3
	Hybrid	20	15.03 ± 0.5
	Hybrid	30	11.15 ± 0.4
Thermal Conductivity (W/m·K)	Hybrid	0	0.115 ± 0.003
	Hybrid	10	0.090 ± 0.004
	Hybrid	20	0.073 ± 0.003
	Hybrid	30	0.085 ± 0.004
Acoustic Attenuation (dB)	Hybrid	0	13.2 ± 0.5
	Hybrid	10	16.4 ± 0.6
	Hybrid	20	20.6 ± 0.7
	Hybrid	30	19.1 ± 0.6
Water Absorption (%)	Hybrid	0	1.9 ± 0.1
	Hybrid	10	3.7 ± 0.1
	Hybrid	20	5.53 ± 0.2
	Hybrid	30	6.2 ± 0.2
Density (g/cm^3^)	Hybrid	0	1.36 ± 0.01
	Hybrid	10	1.30 ± 0.01
	Hybrid	20	1.27 ± 0.01
	Hybrid	30	1.25 ± 0.01

**Table 2:** Weibull shape (η) and scale (β) parameters.

Property	Fiber Type	Content (%)	Shape (η)	Scale (β)	(K-S) *p*
Tensile Strength (MPa)	DPF	0	16.71	18.31	0.882
Tensile Strength (MPa)	DPF	10	15.26	22.11	0.957
Tensile Strength (MPa)	DPF	20	13.11	22.82	0.843
Tensile Strength (MPa)	DPF	30	15.52	21.06	0.850
Tensile Strength (MPa)	Hybrid	0	10.73	17.53	0.809
Tensile Strength (MPa)	Hybrid	10	21.73	21.86	0.995
Tensile Strength (MPa)	Hybrid	20	10.39	28.85	0.947
Tensile Strength (MPa)	Hybrid	30	11.92	23.96	0.651
Fracture Toughness ($MPa\sqrt{m}$)	DPF	0	16.96	3.49	0.985
Fracture Toughness ($MPa\sqrt{m}$)	DPF	10	11.54	7.59	0.953
Fracture Toughness ($MPa\sqrt{m}$)	DPF	20	18.88	12.1	0.924
Fracture Toughness ($MPa\sqrt{m}$)	DPF	30	18.0	9.49	0.830
Fracture Toughness ($MPa\sqrt{m}$)	Hybrid	0	13.2	3.42	0.923
Fracture Toughness ($MPa\sqrt{m}$)	Hybrid	10	16.79	9.34	0.988
Fracture Toughness ($MPa\sqrt{m}$)	Hybrid	20	9.2	15.03	0.856
Fracture Toughness ($MPa\sqrt{m}$)	Hybrid	30	14.3	11.15	0.936

**Table 3:** RSM optimization results and experimental peaks.

Property	RSM Optimization (18.97%)	Experimental Peak (20%)
Tensile Strength (MPa)	25.89	27.00
Toughness ($MPa\sqrt{m}$)	14.23	13.95
Thermal Conductivity (W/m·K)	0.08	0.073
Acoustic Attenuation (dB)	20.49	20.60
Water Absorption (%)	5.11	5.53
Desirability Index	0.673	-

## Data Availability

The data supporting the findings of this study are available from the corresponding author upon reasonable request.
